# The Potential and Application of iPSCs in Gene and Cell Therapy for Retinopathies and Optic Neuropathies

**DOI:** 10.32607/actanaturae.25454

**Published:** 2023

**Authors:** E. V. Lapshin, Y. G. Gershovich, A. V. Karabelsky

**Affiliations:** Gene Therapy Department, Science Center for Translational Medicine, Sirius University of Science and Technology, Krasnodar Region, Sirius, 354340 Russian Federation

**Keywords:** induced pluripotent stem cells, retinopathies, optic neuropathies, retinal ganglion cells, organoids, gene therapy, cell therapy

## Abstract

This review focuses on *in vitro *modeling of diseases and the development of therapeutic strategies using iPSCs for the two most common types of optical pathologies: hereditary neuropathies and retinopathies. Degeneration of retinal ganglion cells and the subsequent optic nerve atrophy leads to various types of neuropathies. Damage to photoreceptor cells or retinal pigment epithelium cells causes various retinopathies. Human iPSCs can be used as a model for studying the pathological foundations of diseases and for developing therapies to restore visual function. In recent years, significant progress has also been made in creating ganglionic and retinal organoids from iPSCs. Different research groups have published data pertaining to the potential of using iPSCs for the modeling of optic neuropathies such as glaucoma, Leber hereditary optic neuropathy, etc., including in the development of therapeutic approaches using gene editing tools.

## INTRODUCTION

In 2007, Takahashi et al. demonstrated that the pluripotent status can be induced in mature somatic fibroblasts by reprogramming through the overexpression of four pluripotent transcription factors (the so-called Yamanaka factors): OCT3/4, SOX2, C-MYC, and KLF4 [1]. Induced pluripotent stem cells (iPSCs) share the morphology of human embryonic stem cells (ESCs) and express their genetic markers.

Overexpression of the transcription factor cocktail makes possible the reprogramming of a patient’s somatic cells to iPSCs [1, 2, 3], which possess such essential characteristics as:

– an ability to differentiate into cells derived from all the germ layers (the ectoderm, the mesoderm, and the entoderm) and

– an unrestricted reproductive capacity while maintaining a normal karyotype, which enables continuous production of the cell material [4, 5, 6].

The advances achieved in the methods that are used to work with human iPSCs have yielded the “disease-in-a-dish” concept. Combining iPSCs and the genome editing technology, analysis of the regulation of metabolic pathways, and phenotype assessment before and after genome editing represent at the moment a powerful tool for studying the progression of optic diseases, including rare inherited retinal disorders, and makes it possible to elaborate methods for testing the efficacy of drugs and novel therapeutic approaches.

Gene therapy is often the only way to manage inherited disorders. Replacement therapy involving correction of the genetic defect by inserting a functional gene copy into a patient’s cells is usually employed. The iPSCs derived from patients’ primary cells are a relevant and convenient model both for *in vitro* screening and for the assessment of the efficacy of gene therapy agents and for predicting the potential adverse effects of the therapy, as well as improving the safety profile of the product.

The genetic material is delivered using cationic polymers, lipid nanoparticles, and different viral vector platforms. Cationic polymers are capable of penetrating into the cell nucleus but can destroy the cell membrane, thus exhibiting a toxic effect on the cell [7]. Lipid nanoparticles encapsulating DNA in liposomes fuse with cell membranes and release genetic material into the cell [8]. The drawbacks of DNA delivery using lipid nanoparticles include low effectiveness, because of the degradation of liposome–DNA complexes by cellular lysosomes. The most commonly used vectors are viruses that, in the case of single administration, ensure efficient delivery and the expression of a therapeutic gene, thus eliciting a longterm response to therapy in patients with severe genetic disorders. The diversity of viral vectors allows one to vary the specificity of their delivery into cells [9]. However, when choosing a viral vector, one should take into account its potential immunogenicity, as well as the risks of insertional mutagenesis that are associated with the application of integrative viral vectors.

The advances achieved in genome editing and the generation of iPSCs have consolidated into the new branch of gene therapy in combination with cell therapy. The technique involving *in vitro *editing of a patient’s pathogenic genotypes and inserting gene-corrected iPSCs for phenotype correction are devoid of the shortcomings of conventional gene therapy, since they ensure immunocompatibility with the recipient and allow one to check the quality of iPSCs prior to transplantation [10].

The main innovation in using iPSCs in gene therapy consists in the development of genome editing approaches employing the CRISPR/Cas9 system and its analogs, whereas there are virtually no studies that deal with gene replacement therapy. The data on the application of iPSCs as models will be presented below, mainly to assess the degree of efficiency in editing autosomal-dominant mutations.

This review focuses on the *in vitro *modeling of the two most common types of disorders of the visual system (hereditary neuropathies and retinopathies), as well as on the development of therapeutic strategies using iPSCs. It also discusses the translational advances in cell and gene therapy.

## GENE AND CELL THERAPY FOR NEUROPATHIES WITH iPSCs

Optic neuropathies caused by retinal ganglion cell death and optic nerve axonal degeneration are the leading causes of vision loss and blindness worldwide [11, 12]. Retinal ganglion cells (RGCs) are specialized neurons, whose axons form the optic nerve transmitting information from the eye to the brain [13]. Glaucoma – a progressive optic neuropathy characterized by structural changes in the optic nerve head (optic disc) and irreversible vision loss – is the most common pathology, diagnosed in more than 60 million people [14, 15, 16, 17, 18, 19]. Other optic neuropathies such as Leber hereditary optic neuropathy (LHON) and autosomal dominant optic atrophy (DOA) manifest themselves at an earlier age and are caused by mitochondrial mutations.

**Gene therapy for neuropathies**

LHON is characterized by the loss of central vision, and it predominantly affects males. Most of the mutations in patients with this disease were uncovered in the mitochondrial genes coding for the proteins of complex I of the electron transport chain (ETC):* MT-ND4 *(m.11778G>A), *MT-ND1 *(m.3460G>A), and* MT-ND6 *(m.14484T>C). The pathogenesis of LHON is associated with decreased ATP synthesis and the accumulation of reactive oxygen species (ROS), leading to retinal ganglion cell death, optic atrophy and, consequently, central vision loss initially in one eye and then in the second eye.

Australian researchers have derived iPSCs from a patient with homoplasmic double mtDNA mutations (m.4160T>C and m.14484T>C) in the *MT-ND1 *and* MT-ND6 *genes, respectively. Such a genotype causes the so-called “Lebers Hereditary Optic Neuropathy Plus” (LHON Plus) disease when additional neurological symptoms, compared to those for optic neuropathy (e.g., movement disorders), develop. Mitochondria in these iPSCs were replaced with non-mutated mitochondria using the cybrid technology. The levels of apoptosis and ROS in RGCs derived from the edited iPSCs were lower than those in the control mutationcarrying RGCs [20].

DOA is the disorder caused by mitochondrial dysfunction presenting as decreased visual acuity at an early age and blindness. RGCs and their axons forming the optic nerve are damaged in patients with DOA. Mutations leading to DOA reside in the *OPA1* gene encoding the inner mitochondrial membrane protein, whose dysfunction affects mitochondrial fusion, ATP synthesis, signaling of apoptosis-inducing factors, calcium metabolism, and maintenance of mitochondrial genome integrity [21].

In iPSCs derived from a patient with the 1334G>A (R445H) mutation in the *OPA1 *gene, the mutation was corrected using CRISPR/Cas9 genome editing combined with homology-directed repair (HDR), with ssDNA used as a template. The oxygen consumption rate (OCR) in the edited iPSCs was higher than that in the mutated cells, which were characterized by reduced mitochondrial fragmentation and a lower level of apoptosis signaling [21].

**Cell therapy for neuropathies**

Cell replacement therapy with iPSCs is a promising approach to the treatment of neuropathies and retinopathies, especially at later stages of the pathologic process, when a significant number of cells have been lost. It is also believed that, in some cases, trophic factors released by stem cells can contribute to regeneration during transplantation. The efficacy of iPSC-based cell therapy has been proved in animal models of optic neuropathy [22, 23]. Ganglion cells differentiated from iPSCs can become integrated and survive after transplantation into the retina of mice used as a disease model [22]. Furthermore, transplantation of iPSC-derived progenitor cells has been shown to promote healing of an optic nerve injury in rats, accompanied by significant prompted potential restoration [23].

Cell therapy for neuropathies can be used to obtain non-ganglion cells. Thus, Abu-Hassan et al. demonstrated that transplantation of iPSC-derived trabecular meshwork cells can restore the homeostatic function in an *ex vivo *human anterior segment perfusion culture model [24], thus opening an interesting novel approach to the treatment of glaucoma.

Currently, there are no reports on clinical trials of cell therapy for optic neuropathy, but clinical trials of replacement retinal pigment epithelium cells derived from iPSCs are being conducted [25, 26]. The Advanced Cell Technology stem cell company has recently reported that phase I/IIa clinical trials of a suspension of retinal pigment epithelium cells derived from ESCs transplanted to patients with age-related macular degeneration and Stargardt disease have been successfully completed [25, 26]. Vision was improved, and neither serious adverse events nor immune responses were observed after low-dose transplantation of cells differentiated from ESCs into one eye in 18 patients. No data on rejection of the transplanted cells, uncontrolled cell proliferation, or serious eye or systemic problems have been reported. Visual functions were improved in most patients, and the target safety endpoints were attained in the trials. Furthermore, the team led by Prof. Masayo Takahashi (Japan) is preparing to launch clinical trials using iPSC-derived retinal pigment epithelium to treat agerelated macular degeneration [26]. These clinical trials will confirm the conceptual feasibility of using pluripotent stem cells to restore the functionality of affected tissues, thus offering a new option for effective and safe treatment of blindness caused by different pathological processes.

## GENE AND CELL THERAPY FOR RETINOPATHIES WITH iPSCs

Inherited retinopathy is defined as any genetic disorder leading to retinal damage and, therefore, visual impairment. The prevalence of diseases belonging to this group is approximately 3 out of 100 people. The most common symptoms of retinopathies include visual field defects, an inability to adapt to poorly illuminated environments, distortion of objects’ shape and size, as well as altered color perception. The data on the molecular processes associated with these diseases have been mostly acquired from fibroblast models, since retinal samples cannot be obtained. The use of iPSCs for this purpose can yield a more relevant model of the disease.

According to their type, retinopathies can be macular or peripheral. The central part of the retina (the macula) is affected in patients with macular retinopathy (e.g., Stargardt and Best disease). Peripheral vision is impaired in patients with peripheral retinopathy. The most common diseases belonging to this group include retinitis pigmentosa and choroideremia [27].

Different types of Leber congenital amaurosis (LCA) causing vision loss at birth or soon after are believed to be the most severe and earliest forms of inherited retinal disorders. Patients with this disease may also develop light hypersensitivity, involuntary eye movements (nystagmus) and farsightedness. Mental retardation can be observed in rare cases.

**Application of gene therapy approaches to the treatment of inherited retinopathies**

There are at least 20 types of LCA that are caused by different mutations in various genes, as well as by phenotypic manifestations. The most common pathological mutations in patients with LCA include mutations in the *CEP290*, *CRB1*, *GUC2D*, and *RPE65 *genes. The molecular genetic reasons for LCA have yet to be identified in ~ 30% of cases [28]. Thus, the protein encoded by the *CEP290 *gene is involved in cell division, microtubule assembly, as well as the formation of centrosomes and cilia. Mutations in this gene cause the most severe form of LCA: LCA type 10 [29]. Up to 15% of all the *CEP290 *mutations are represented by the IVS26 – 2991+1655 A>G mutation in intron 26, which leads to the insertion of exon carrying a stop codon (C998X). The truncated peptide resulting from this mutation ensures only partial activity of CEP290. The iPSC model derived from a patient with this genotype was edited using the CRISPR/Cas9 sys tem. Compared to mutations in the coding region of the gene requiring a recombination template, splice site mutations can be corrected through targeted deletion. Thus, genome editing in iPSCs involving deletion of the splice site in the IVS26 region increased the synthesis level of functional CEP290 in [29].

Gene therapy using non-coding RNA targeting the IVS26 mutation proved to be effective in a 3D retinal organoid model derived from a patient’s iPSCs. A fully phosphorothioate-modified and 2’-O-methylmodified RNA oligonucleotide (QR-110) corrected the CEP290 splicing defect and restored the wild-type mRNA. Dose-dependent restoration of photoreceptor cilia was demonstrated [30].

LCA type 4, caused by *AIPL1 *mutations, is characterized by severe vision impairment during infancy and progressive photoreceptor atrophy. The retinal organoid derived from the iPSCs of a patient carrying the 834 G>A (Trp278X) mutation in the *AIPL1 *gene was edited using the CRISPR/Cas9, combined with HDR approaches with a 30% effectiveness. *AIPL1 *expression was restored after editing, and the cGMP and PDE6 levels in the cells increased [31].

LCA type 7, which constitutes about 2% of all LCAs, is characterized by early photoreceptor dysfunction caused by mutations in the *CRX *gene (encoding the cone-rod homeobox protein). NHEJ (nonhomologous end joining)-mediated CRISPR/Cas9 editing of the 263A>C (K88Q) mutation in the *CRX* gene in the retinal organoid model contributed to the development and maturation of photoreceptor cells. Interestingly, the genome editing strategy involved the insertion of two double-strand breaks. One of them targeted the mutation, while the other one targeted the allele-specific SNPs between exons 2 and 4 of the *CRX* gene [32].

Retinitis pigmentosa (RP) is an inherited disease affecting the retina and characterized by progressive photoreceptor loss. Patients experience problems with night and peripheral vision, although total blindness is quite rare. The disease onset usually takes place in childhood. One of the possible causes of RP is a mutation in the rhodopsin (*RHO*) gene [33]. Rhodopsin, a visual pigment found in the retinal rods, is a transmembrane receptor bound to G proteins; its conformation changes upon absorption of light quanta. Rhodopsin activates the G protein transducin, which activates cGMP-dependent phosphodiesterase, further enducing the permeability of cGMP-dependent ion channels, membrane hyperpolarization, and the generation of a nerve impulse [34]. By using a helper-dependent adenoviral vector (HDAdV), the editing of the mutation in the iPSCs of a patient carrying the mutation causing E181K substitution in the rhodopsin molecule was performed. The edited iPSCs differentiated into photoreceptor cells were characterized by a decreased level of autophagy due to the suppression of ER stress-induced apoptosis. HDAdV gene transfer was performed by homologous recombination without the insertion of DNA breaks [34].

The 68C>A (P23H) mutation in this gene was also successfully edited using the CRISPR/Cas9 system in the iPSC model. No nonspecific gene editing was observed in wild-type (control) cells, while in mutant cells editing resulted in frameshift and translation termination, causing the inactivation of the mutant allele [29].

X-linked retinitis pigmentosa affects males (with an incidence of 1 case for every 15,000 individuals) and manifests itself as impaired night vision followed by a loss of peripheral vision and total blindness by age 40. In this case, mutations reside in the *RPGR *gene encoding the retinitis pigmentosa GTPase regulator, which affects the development of photoreceptor cells, a component of the centrosome– cilium protein interaction landscape. Approximately 16% of RP cases are associated with mutations in the* RPGR *gene. By using the CRISPR/Cas9 and HDR approaches, gene editing of iPSCs derived from a patient carrying the 3070 G>T mutation in the* RPGR *gene, where the single-strand template was mutation-free, was performed. Although this gene is GC-rich and carries nucleotide repeats, the editing efficiency amounted to 13% [35]. Deletions in *RPGR* exon 14 resulting in frameshift and loss of the sequences encoded by exons 15–19 are known. Such mutations impair ciliogenesis; therefore, patients with this defect have shortened photoreceptor cilia. The iPSCs derived from patients with *RPGR *mutation variants (1685_1686delAT, 2234_2235delGA, and 2403_2404delAG) were edited with the CRISPR/Cas9 tool, combined with HDR. The resulting three-dimensional retinal organoids had normal morphology, expressed recoverin, and contained a larger number of rods and cones compared to the control [36].

X-linked juvenile retinoschisis characterized by degenerative neuropathy and retinal detachment is another X-linked disorder. Juvenile retinoschisis develops predominantly in males; its incidence is approximately 1 case for 10,000 individuals. This disease is caused by mutations in the *RS1 *gene involved in the cellular organization of the retina and intercellular adhesion. The iPSC models were derived from patients carrying the 625C>T (R209C) and 488G>A (W163X) mutations. The editing efficiency for iPSCs edited using the CRISPR/Cas9-mediated HDR approach amounted to 50%, but insertions were also present. For the 625C>T mutation, the efficiency of Cas9-ABE7.10-mediated base editing was comparable to that achieved using the HDR approach [37].

Mutations in the *PRPF *genes causing RP type 13 are autosomal dominant and are observed in ~ 15% of all retinitis pigmentosa cases. The protein encoded by the *PRPF8 *gene plays a crucial role in pre-mRNA splicing. It is the major component of the U2-type or U12-type spliceosome, and it is responsible for spliceosome positioning on pre-mRNA. iPSCs carrying the 6901 C>T (P2301S) mutation in the *PRPF8 *gene were edited using the CRISPR/Cas9-Gem (Cas9 endonuclease and heminin protein) system via HDR. The edited iPSCs differentiated into retinal epithelial cells and regained morphology and apical–basal polarity, as well as the ability to phagocytize photoreceptor outer segments. Cas9-Gem was used for system degradation during the G0/G1 phase to reduce the probability of NHEJ-mediated insertions [38].

CRISPR/Cas9 genome editing, combined with HDR, was used to edit iPSCs derived from a patient carrying the 1115_1125del11 mutation in the *PRPF31* gene encoding the component of the pre-mRNA spliceosome complex (retinitis pigmentosa type 11). This gene editing restored the molecular and cellular phenotypes of the induced retinal organoids [39].

The *MERTK *gene, whose mutations cause autosomal recessive retinitis pigmentosa, encodes receptor tyrosine kinase transmitting signals from the extracellular matrix to the cytoplasm. This enzyme is involved in cell differentiation, cell survival, and phagocytosis of apoptotic cells. The 992_993delCA mutation in the *MERTK *gene was corrected in patient-derived iPSCs using CRISPR/Cas9 genome editing, combined with HDR. The edited iPSCs differentiated into retinal pigment cells and restored *MERTK *expression and phagocyte functions, compared to those observed in mutant variants [40, 41].

The main cause of recessive retinitis pigmentosa in ethnic Jews is a 354-bp Alu insertion in the *MAK* gene encoding serine/threonine protein kinase that is involved in cell cycle regulation and is important for the regulation of the cilium length and photoreceptor cell survival. CRISPR/Cas9-mediated editing of iPSCs via the HDR approach involving Alu insertion restored the *MAK *transcript [29].

Enhanced S-cone syndrome is caused by a mutation in the *NR2E3 *gene encoding the transcription factor activating rod development and suppressing cone development. Patients with this syndrome typically suffer from retinal atrophy, followed by loss of vision. S cones belong to one of the three types of eye cones that is the least abundant in a normal human retina. Mutations in the *NR2E3 *gene result in differentiation defects, accompanied by the formation of a large number of S cones and the absence of rods. CRISPR/Cas9- and NHEJ-mediated mutant allele knockout in iPSCs derived from a patient carrying the 166G>A (G56R) mutation in the *NR2E3 *gene resulted in normal functioning and development of rod photoreceptors in differentiated retinal organoids [42].

Usher syndrome is a disease that causes a loss of vision in late stages (as a result of retinitis pigmentosa) and hearing loss in earlier stages; vestibular disorders are also possible. One of the causes of this disease is a mutation in the *MYO7A *gene encoding myosin, the retinal motor protein that is involved in the renewal of the photoreceptor outer segment discs, contributes to the distribution and migration of the melanosomes and phagosomes in retinal pigment epithelium, and is associated with the regulation of opsin transport in retinal photoreceptors. iPSCs derived from a patient with *MYO7A *mutations (c.1184 G>A and c.4118C>T) were subjected to CRISPR/Cas9 genome editing, combined with HDR. Morphological (as stereocilia adhesion) and functional (as restoration of membrane potential) recovery of differentiated edited hair cells was then observed [43]. The Usher syndrome is also associated with mutations in the *USH2A *gene encoding the usherin protein, which is involved in sound and light perception as a member of the USH2 complex. In retinal photoreceptors, the USH2 complex supports the periciliary membrane complex, which plays a role in the regulation of intracellular protein transport. Patient-derived iPSCs were subjected to CRISPR-eSpCas9 genome editing, combined with HDR, to correct the 2276G>T (C759F) and 2299delG (E767Serfs*21) mutations located 22 bp apart from *USH2A *exon 13. A 15% editing efficiency and restoration of *USH2A *gene expression was been achieved. Moreover, iPSCs retained their genomic stability and pluripotency [44, 45].

**Cell therapy for retinopathies**

The iPSC-based cell therapy has proved to be effective in animal models of retinopathies. Thus, the human iPSC-derived retina was transplanted into the subretinal space in monkeys with laser-induced retinal injury and in immunodeficient rats with retinitis pigmentosa. The transplanted cells were integrated into the rat retina to form synaptic connections with host bipolar cells. In the monkey model, the transplanted cells integrated into the host retina; improvement of electroretinogram (ERG) was also recorded [46]. In a similar manner, in the mouse model of retinitis pigmentosa, subretinal transplantation of iPSC-derived retinal spheroids delayed retinal thinning, increased the level of pigment epithelium-derived factor (PEDF), and reduced the number of apoptotic cells and the level of microglial infiltration into the retina [47]. In rats with an inherited mutation in the MER proto-oncogene tyrosine kinase (*MERTK*) gene as a model of retinal degeneration, subretinal transplantation of iPSC-derived RPE cells significantly restored the visual function as measured by thresholds in optokinetic tracking. None of the animals showed abnormal proliferation or teratoma formation [48]. Interestingly, co-transplantation of different types of retinal cells derived from iPSCs showed better results compared to the transplantation of individual cell types. This resulted in a better visual response and preservation of the outer nucleolar layer in the retinal degeneration rat model [49]. In the animal model of retinitis pigmentosa, subretinally transplanted iPSC-derived photoreceptor precursors expressing *CRX *were incorporated into the inner nuclear layer of cells. The transplanted cells expressed the marker arrestin 3, which was indicative of their further maturation [50].

In the preclinical study in rats and pigs, after differentiation into retinal cells, iPSCs derived from the CD34+ cells of patients with macular dystrophy integrated and restored the retina. This study revealed that 10-fold fewer cells were required during monolayer transplantation to attain the therapeutic effect than when using a cell suspension. Meanwhile, retinal cells transplanted as a suspension failed to integrate into the retinal ganglion cell layer of the rat; the poly(lactic-co-glycolic acid) (PLGA)-based scaffold facilitated the integration of the transplanted cell layer into the Bruch’s membrane of the rat [51].

## CONCLUSIONS

**Fig. 1:**
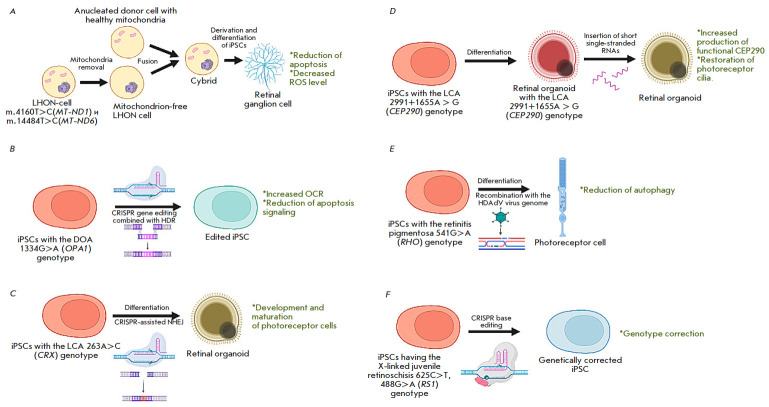
Gene therapy approaches to the treatment of hereditary retinopathies/neuropathies in iPSC models. (*A*) – The mitochondrial replacement approach, creating cybrids in the LHON model. (*B*) – CRISPR editing combined with HDR in the DOA model. (*C*) – CRISPR editing with NHEJ in the LCA model. (*D*) – RNA interference in the LCA model. (*E*) – Recombination with the HDAdV genome in the retinitis pigmentosa model. (*F*) – CRISPR base editing using ABE7.10 in the model of X-linked juvenile retinoschisis

Although the application of iPSCs in studies devoted to optic neuropathies and retinopathies is a relatively new approach, this technology undoubtedly has a high potential in terms of investigating the pathogenesis of diseases, as well as validating and optimizing gene therapy and genome editing technologies (*Fig. 1*, *Table 1*). Disease modeling using iPSCs allows one to study the main mechanisms causing loss of retinal ganglion cells; cell replacement therapy using iPSCs derived from the patient’s own somatic cells presents a minimal risk of immune rejection after transplantation and exhibits high efficacy for different models. Gene therapy, in combination with cell replacement therapy, can be used to correct genetic defects in iPSC-derived cells prior to transplantation.

iPSCs have a tremendous translational potential in a broad range of therapeutic areas. The development and improvement of protocols for enhancing the efficacy and purity of iPSC-derived retinal ganglion cells will be critical in elaborating a standardized methodology for using iPSCs in disease modeling, drug screening, toxicology studies, cell and gene therapy, as well as regenerative medicine.

**Table 1:** Studying the potential of gene therapy approaches and gene editing of inherited retinopathies and optic neuropathies using the iPSCs models

Disease	Mutation	Inheritance type	Treatment approach	Main effects/outcome
LHON^*1^	m.4160T > C(MT-ND1) and m.14484T > C(MT-ND6)	Maternal, mitochondrial	mitochondrial replacement, cybrid generation	Reduction of apoptotic effects and ROS level^*2^ in differentiated RGCs^*3^ [20]
DOA^*4^	1334G > A (OPA1)	Autosomal dominant	CRISPR genome editing combined with HDR^*5^	Increased OCR^*6^ in edited iPSCs^*7^ and reduction of apoptotic signals [21]
LCA^*8^	2991+1655A > > (CEP290)	Autosomal recessive	CRISPR-assisted NHEJ^*9^, RNA interference	Increased production of functional CEP290. Restoration of photoreceptor cilia [29, 30]
	834G > A (AIPL1)	Autosomal recessive	CRISPR genome editing combined with HDR	Restoration of AIPL1 gene expression, increased cGMP^*10^ and PDE6 levels in retinal organoid cells [31]
	263A > C(CRX)	Autosomal recessive	CRISPR-assisted NHEJ	In the model of retinal organoids derived from patients’ iPSCs, promoted the development and maturation of photoreceptor cells [32]
Retinitis pigmentosa	541 G>A (RHO)	Autosomal dominant	Recombination with the HDAdV genome	After gene editing, iPSCs differentiated to photoreceptor cells exhibited reduced autophagy [34]
	68C>A (RHO)	Autosomal dominant	CRISPR-assisted NHEJ	Inactivation of mutant allele [29]
	3070G>T (RPGR)	X-linked	CRISPR genome editing combined with HDR	Restoration of the nucleotide sequence [35]
	1685_1686delAT, 2234_2235delGA and 2403_2404delAG (RPGR)	X-linked	CRISPR genome editing combined with HDR	Retinal organoids had a normal morphology [36]
	6901C>T (PRFP8)	Autosomal dominant	CRISPR genome editing combined with HDR	The morphology and phagocytizing ability were restored in edited iPSCs differentiated into retinal epithelial cells [38]
	1115_1125del11 (PRPF31)	Autosomal dominant	CRISPR genome editing combined with HDR	Restoration of the molecular and cellular phenotypes in induced retinal organoids [39]
	992_993delCA (MERTK)	Autosomal recessive	CRISPR genome editing combined with HDR	Restoration of MERTK gene expression and phagocytic function [40, 41]
	354-bp Alu insertion (MAK)	Autosomal recessive	CRISPR genome editing combined with HDR	Restoration of MAK transcript [29]
X-linked juvenile retinoschisis	625C > T, 488G > A (RS1)	X-linked	CRISPR-mediated base editing using the ABE7.10 system	Restoration of the nucleotide sequence [37]
Enhanced S-cone syndrome	166G > A (NR2E3)	Autosomal recessive	CRISPR-assisted NHEJ	Normal functioning and development of rod photoreceptors in differentiated retinal organoids [42]
Usher syndrome	c.1184G > A and c.4118C > T (MYO7A)	Autosomal recessive	CRISPR genome editing combined with HDR	Morphological (stereocilia adhesion) and functional recovery (restoration of the membrane potential) [43]
	2276G > T (USH2A)	Autosomal recessive	CRISPR genome editing combined with HDR	Restoration of the nucleotide sequence [44, 45]

^*1^ – Leber hereditary optic neuropathy;

^*2^ – reactive oxygen species;

^*3^ – retinal ganglion cells;

^*4^ – autosomal dominant optic atrophy;

^*5^ – homology-directed repair;

^*6^ – oxygen consumption rate;

^*7^ – induced pluripotent stem cells;

^*8^ – Leber congenital amaurosis;

^*9^ – non-homologous end joining;

^*10^ – cyclic guanosine monophosphate.

## Abbreviations

CRISPR: clustered regularly interspaced short palindromic repeats
RGCs: retinal ganglion cells
POG: primary open-angle glaucoma
IOP: intraocular pressure
LHON: Leber hereditary optic neuropathy
DOA: autosomal dominant optic atrophy
AMD: age-related macular dystrophy
DR: diabetic retinopathy
RP: retinitis pigmentosa
ESCs: embryonic stem cells
iPSCs: induced pluripotent stem cells
hESCs: human embryonic stem cells
ERG: electroretinogram
ETC: electron transport chain
HDR: homology- directed repair
NHEJ: non-homologous end joining
ROS: reactive oxygen species
OCR: oxygen consumption rate
LCA: Leber congenital amaurosis
